# A patient satisfaction survey investigating pre- and post-operative information provision in lower limb surgery

**DOI:** 10.1186/s12891-020-03761-w

**Published:** 2020-11-16

**Authors:** Maxwell Stanley Renna, Andrew Metcalfe, David Ellard, David Davies

**Affiliations:** 1 Warwick Medical School, Medical School Building, Coventry, CV4 7HL UK; 2 Trauma & Orthopaedics, University Hospitals Coventry & Warwickshire, Coventry, UK; 3 WMS - Warwick Clinical Trials Unit, Warwick Medical School, Coventry, UK; 4 WMS - Social Science and Systems in Health, Warwick Medical School, Coventry, UK

**Keywords:** Patient satisfaction, Lower limb surgery, Survey, Experience, Recovery

## Abstract

**Background:**

Planned lower limb surgery is common, with over 90,000 hip replacements, 95,000 knee replacements and 15,000 anterior cruciate ligament reconstructions performed in the UK each year. These procedures are primarily indicated to treat osteoarthritis, sporting injuries and trauma. Patient satisfaction is an important element of healthcare provision, which is usually measured by functional outcomes but influenced by other factors. Few studies have assessed patients’ views on the information given to them pertaining surgery and patients are infrequently consulted when designing leaflets and information packs, which can lead to confusion during the recovery period and poor long-term outcomes. Furthermore, previous studies have not directly asked patients what resources they would prefer, or which format would suit them best. This project aimed to assess if patients were satisfied with the information they received around their operations and to identify potential improvements.

**Methods:**

Set in a National Health Service (NHS) run major trauma centre in the West Midlands, a multiple choice and free-text answer survey was administered to patients who used the orthopaedic service over the course of 1 month. Surveys were designed in Qualtrics and administered face-to-face on paper. Thematic content analysis was performed on the results.

**Results:**

Eighty patients completed the survey, of which 88.8% of patients were satisfied with the information they received. Discussions with surgeons were the most useful resource and 53% of patients requested more internet resources. Post-operative patients were statistically more likely to be dissatisfied with information provision than pre-operative patients. Over 20% of the study population requested more information on post-operative pain and recovery timelines.

**Conclusions:**

Although patients were satisfied in general, areas for change were identified. Suggested resources took the form of webpages and mobile platforms. These resources could contain educational videos, patient experience blogs or interactive recovery timelines, to be of benefit to patients. These suggestions may enable NHS Trusts to “get into the digital age”, however, more research on patient satisfaction around information provision and the impact it has on recovery and decision making is needed.

**Supplementary Information:**

The online version contains supplementary material available at 10.1186/s12891-020-03761-w.

## Background

Planned lower-limb operations, such as knee and hip replacement, can be very effective treatments which are becoming increasingly commonly used [1–3]. These procedures are primarily used for Osteoarthritis (OA), a common degenerative condition of the joints, which is increasing in prevalence due to ageing populations and the obesity epidemic [4]. Over 92,000 hip and 98,000 knee arthroplasty operations were performed across the UK in 2018, an increase of 53 and 37% respectively since 2007 [5]. These numbers have been predicted to quadruple by 2035 [6]. However, patient dissatisfaction after knee arthroplasty is high, estimated to range between 8 and 17% [7].

Non-joint replacement operations are also common in the lower limb. It is estimated that over 15,000 Anterior Cruciate Ligament Reconstruction (ACLR) operations are performed in the UK each year [8]. Additionally, approximately 40,000 meniscal injuries requiring meniscectomy occur in the UK per year [9]. Traditionally, these injuries have been caused by traumatic sporting events and have been associated with future onset of OA, further increasing the burden of joint replacement services [10, 11]. These procedures have also been impacted by overly optimistic expectations, which may lead to long-term dissatisfaction with their outcomes [12].

Previous studies have measured satisfaction as well as functional outcomes or Patient Reported Outcomes (PROs) in lower limb surgery, concluding that three elements are key in maintaining high levels of satisfaction. These include meeting preoperative expectations, adequate pain relief and the patient’s subjective hospital experience [13, 14]. The strongest predictor of dissatisfaction after total knee replacements or ACLR procedures is the failure to meet patients’ preoperative expectations [12]. This demonstrates a lack of information being passed on the patient or a lack of understanding of the information being given. Thus, preparing patients more effectively before surgery may provide better satisfaction and functional outcomes [15].

Measuring outcomes has become an increasingly important tool in the field of medicine, however satisfaction largely remains an undefined and difficult to measure parameter, especially regarding information provision within a clinical setting [16]. Whilst PROs are established healthcare quality measurements, patients are sometimes disappointed that questionnaires often do not ask them the right questions [17]. Even though evaluators use satisfaction as a single measure to reflect quality, satisfaction is multifactorial and requires capturing patient views more comprehensively than merely through simple scales, which are frequently used in surveys [18]. To fully capture the patient experience, open ended questions and more detailed assessments are needed to enable patients to offer a holistic view on the information and care they received [16]. This project’s uniqueness stems from directly asking patients which resources they would prefer and in which format these would be best received, an aspect that the aforementioned studies have neglected.

It has been suggested that by ensuring adequate patient education and realistic goal setting, patient satisfaction could be improved [19, 20]. Additionally, with the WHO’s global strategy on people-centred care and integrated health models becoming more prevalent, a simple yet effective method of ensuring the patient is at the heart of clinical decisions must be identified and utilised. Healthcare professionals must provide relevant, effective and tailor-made information in the medium best suited to or requested by the patient, to ensure they are able to make the most informed decision possible [21]. To achieve both these targets, we must first ascertain if patients are satisfied with the information they receive, pertaining their operations.

This project is set in a National Healthcare Service (NHS) run tertiary orthopaedic centre in the West Midlands that provides free at the point of care services and is currently rated “good” by the Care Quality Commission [22]. At the moment, within the Trust assessed, patients receive a consultation with a surgeon, an appointment with a physiotherapist prior to their operation, and a leaflet relevant to their operation. However, patients have not been involved in the production of the leaflets. Patients undergoing joint replacement procedures will be invited to a group session, with other patients, to discuss exercises and extra support options with a trained nurse practitioner. Post-operatively, all patients are given further appointments with their surgeon at 6 weeks, 6 months and 1 year. They are also given six weekly sessions with a physiotherapist and a telephone number to call in case they have any issues. Currently, patients do not have any electronic resources provided by the Trust and although every effort is made to provide patients with a robust and individualised experience, cost restrictions and workforce limitations hinder the development and implementation of such resources. Electronic resources such as educational videos and an app based post-operative educational system are currently in development but have not been completed. This project may therefore help guide what should be included within these, to meet the needs of the study population. The project also aims to assess whether sub-groups of the patient population require different resources, to propose ways of achieving more effective information provision within this lower limb surgical department.

## Methods

### Study design

The study utilised a patient survey, designed by the team with input from expert patients and experienced healthcare professionals alongside current practices and procedures (see supplementary information). It included both closed and open-ended questions and was administered face-to-face on paper to patients attending the orthopaedic department at a major trauma centre in the West Midlands (UK).

### Questionnaire design

The questionnaire was designed in Qualtrics (Qualtrics International Inc., Utah, U.S.) and had three parts entitled demographics, pre-op questions and post-op questions. Demographic data was collected to identify if certain characteristics could be related to the level of satisfaction or information needs. Identical questions were given to both pre- and post-operative patients where possible. In some instances, changes to question wording was required. Where similar questions and responses were recorded, they were amalgamated during analysis. Open-ended questions were used to give the opportunity for patients to write their own suggestions, guided by previous literature. Questions were structured in various styles including tick boxes, matrixes and free-text boxes. Satisfaction was scored by the patients using a Likert scale with five options. Paper copies of the questionnaire were given to patients and was the preferred method of data collection as poor mobile signal in hospital sites made online questionnaires impracticable.

### Sample

Patients were identified by the referring surgeon, contacted by the researcher and invited to participate in the survey on the day of their procedure or during a follow-up appointment. Patients were given a patient information leaflet informing them of how to take part as well as the risks and benefits of doing so and a contact in case of queries or problems. They were informed that completion of the questionnaire and returning it was taken as them consenting to the study, they were also given an information leaflet containing details of data use and how to withdraw. Convenience based sampling was used, whereby patients who turned up during the data collection timeframe and accepted were to be included in the analysis. The survey was anonymous. No patient identifiable data were collected and so patients could not withdraw their data after completing the survey.

### Inclusion criteria

Patients must have had, or be on the waiting list for, lower limb surgery. Example procedures included, but were not limited to, joint replacements, anterior cruciate ligament repairs, meniscal repairs or labral repairs. Patients were excluded if they had to undergo a non-elective surgical procedure. Post-operative patients must have been within 1 year of having their operation.

### Data collection, storage and analysis

The results of the surveys were transcribed by the researcher to the Qualtrics database. At the end of the four-week data collection period, the results data were downloaded, analysed and any hardcopies shredded. The questionnaire was piloted prior to wider use. Questions that had similar themes were aggregated if possible and scores of “extremely satisfied” or “satisfied” were counted as “satisfied”, with “neither satisfied or dissatisfied”, “dissatisfied and “extremely dissatisfied” counted as dissatisfied with the service received. Quantitative data was analysed in SPSS (V26, IBM, New York, U.S.) and relevant statistical tests were performed. Where percentages are given in the results section, this demonstrates the number of respondents who ticked the associated box. Free text boxes were included within the questionnaire and a thematic content analysis was performed on these responses whereby qualitative data was coded in themes relating to the question asked using NVivo (V12, QSR International, Melbourne, Australia). During survey administration, field notes were made, included within the patients’ responses and themed accordingly during analysis.

Statistical analysis included Chi-squared testing. Where a value within the table was less than five, Fisher’s exact tests were performed. These parametric tests were chosen to adequately assess the equality of fit and proportional distribution between categories to identify statistically significant results. In this exploratory analysis, multiple testing corrections were not employed. Therefore, the statistical analysis should be interpreted as exploratory in nature and not confirmatory.

## Results

### Demographics

Eighty patients filled out a questionnaire, with 35 (44%) filling in the pre-op section and 45 (56%) filling in the post-op section. Overall, 71 out of 80 patients (88.8%) indicated they were satisfied or extremely satisfied with the information they had received pertaining their operation. Demographic information can be seen in Table 1. There were no statistically significant characteristics that influenced satisfaction. Forty-one of 47 (87%) patients undergoing joint replacement therapy were satisfied with the information they received and 30 of 33 (90.9%) patients undergoing reparative procedures indicated similar percentages. Patients were statistically significantly more likely to be satisfied with the information provision pre-operatively (*P* = .036, Fisher’s exact test) and post-operative patients were less likely to be satisfied with the care they were provided (*P* = .004, Fisher’s Exact test).

**Table 1:** Demographic characteristics of patients and their satisfaction scores

	Demographic characteristics	No. Patients ***n*** = (%)	Satisfied ***n*** = (%)	Dissatisfied ***n*** = (%)
**Gender**	Male	43 (53.8)	40 (93.0)	3 (7.0)
	Female	37 (46.3)	31 (83.8)	6 (16.2)
**Age (years)**	30 or less	12 (15.0)	10 (83.3)	2 (16.7)
	30–50	24 (30.1)	23 (95.8)	1 (4.2)
	50–70	29 (16.2)	27 (93.1)	2 (6.9)
	70 or more	15 (18.8)	11 (73.3)	4 (26.7)
**Procedure**	TKR	31(39)	27 (87.1)	4 (12.9)
	THR	16 (20)	14 (87.5)	2 (12.5)
	ACLR	14 (18)	12 (85.7)	2 (14.3)
	Meniscal reconstruction^a^	8 (10)	8 (100.0)	0 (0.0)
	HTO	5 (6)	5 (100.0)	0 (0.0)
	Other	6 (8)	5 (83.3)	1 (16.7)
**Operative status**	Pre-op	35 (43)	34 (97.1)	1 (2.9)
	Post-op	45 (56)	37 (82.2)	8 (17.8)^b^
**All Patients**		80 (100)	71 (88.8)	9 (11.3)

*TKR* Total knee replacement, *THR* Total hip replacement, *ACLR* Anterior cruciate ligament reconstruction, *HTO* High tibial osteotomy

^a^Includes meniscectomies, ^b^statistically significant result

### Usefulness of resources

Patients were asked to rate the usefulness of specific resources they were given before their operation. These results can be seen in Table 2.

**Table 2:** Resources found most/least useful and the mean response, rounded to 3 significant figures

Resource (***n*** = 80)	Useful ***n*** = (%)	Not useful ***n*** = (%)	Did not use/ receive ***n*** = (%)
**Leaflet**	58 (72.5)	10 (12.5)	12 (15.0)
**Internet Pages**	18 (22.5)	12 (15.0)	50 (62.5)
**Discussion with surgeon**	68 (85.0)	6 (7.5)	6 (7.5)
**Friends and family**	45 (56.3)	17 (21.3)	18 (22.5)
**Mean**	47.3 (59.1)	11.3 (14.1)	21.5 (26.9)

Sub-group analysis indicated three significant results. Friends and family (patients with similar operative experiences) were significantly more useful when consulted for reparative procedures as opposed to joint replacement X^2^ (3, *n* = 80) = 8.16, *P* = .017. They were also deemed to be more useful by post-operative patients X^2^ (2, *n* = 80) = 17.52, *P* < .001. Leaflets were significantly more useful in post-operative patients than pre-operative ones X^2^ (2, *n* = 80) = 10.02, *P* = .007.

### Resource medium

Before their operations, patients were asked what internet resources, if any, they had consulted. Of the 35 pre-op patients, 14 (40%) had looked at an “NHS website” and five (14%) had visited “WebMD”. They were also asked what resources they would have wanted to have access to before their operation, if they were to undergo it again, results can be seen in Table 3. Twenty pre-operative patients (57%) suggested they would have liked to have had a one to one with a patient who has previously undertaken the operation, however three suggested that discussions with non-healthcare individuals were negative experiences not be beneficial. Four patients also proposed exposure to patients who could give them a balanced reflection of their experiences. Two patients suggested that blogs would be useful for them.

**Table 3:** Frequency, percentage of answers and free-text responses related to suggestions

**Answer (*n*** **= 35)**		***n*** **= (%)**
**1 to 1 with another patient who has had the procedure**		20 (57)
**Leaflets**		19 (54)
**1 to 1 with a healthcare professional**		19 (54)
**Group session with other patients**		16 (46)
**Internet page**		15 (43)
**App on phone**		13 (37)
**Frequent free-text responses (*n*** **= 23):**		
**Internet**	Positive (*n* = 4)	*“Most of the information found out about recovery was from webpages, including YouTube” – Pt 30* *“Used internet to find out about surgeon and surgery. Videos for condition and pathophysiology” – Pt 20*
	Negative (*n* = 3)	*“Internet resources were very gory, too much information for me” – Pt 7* *“[The] internet had lots of information and confused me, especially the American sites.” – Pt 11*
**Friends and family**	Positive (*n* = 6)	*“Learnt from others about how to recover and what to expect” – Pt 32* *“[My] Friend’s husband had one some time ago and told me how good it was, he still golfs and bowls” – Pt 8*
	Negative (*n* = 3)	*“Chatting with others gave me conflicting ideas that might not apply directly to my circumstances” – Pt 33*

Question asked: “We have thought of a few ways to improve the service we provide, please tick those that would help you. If you had the procedure again”. Patient numbers and percentages are included next to their quote. Includes only pre-operative patients

**Fig. 1:**
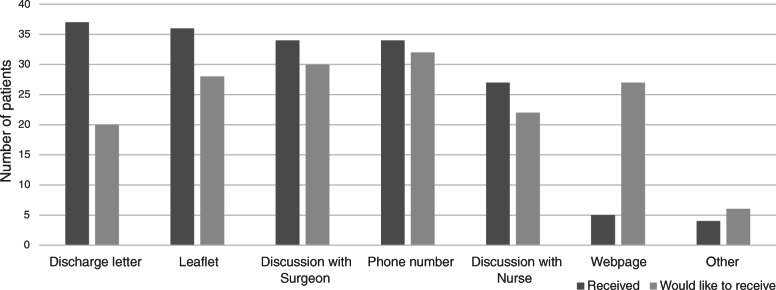
Received vs requested methods of receiving information for post-operative patients (*n* = 45)

Post-op patients were asked by what means they received information after their operation and then how they would they have liked to receive information, see Fig. 1 for results. In total, 42 out of 80 (53%) patients requested more internet resources. This was also reflected in free text responses, with the majority requesting for online recovery timelines and videos.

### Additional information requests

All patients were asked if they were satisfied with specific topics relating to their surgery, seen Table 4. Sixty five of the 80 patients (81%) identified at least one topic they wanted to have received more information on.

**Table 4:** Patient requests for more information on specific topics, with common free text responses below

**Topic (*n*** **= 80)**		***n*** **= (%)**
Post-Operative Pain		22 (27.5)
How long to recover		20 (25.0)
How long before they can work		16 (20.0)
Who will be in the operating room with the patient		8 (10.0)
Pain medication		8 (10.0)
Support available		7 (8.8)
The procedure		6 (7.5)
How to care for wounds		6 (7.5)
**Frequent free-text responses (*n*** **= 47):**		
**Recovery**	*n* = 16	*“More information on recovery timelines and milestones” – Pt 38* *“Specific restrictions -* e.g. *can I ever do breaststroke or lean over?” – Pt 78* *“Medium to long term rehab - information dropped off after the first few weeks” – Pt 69*
**Procedure**	*N* = 11	*“How long would the surgery take?” – Pt 22* *“What sort of hip it will be. (material)” – Pt 8* *“How long the scar will be and where specifically it will be.” – Pt 7*
**Physiotherapy**	*n* = 5	*“More physio exercises/videos in the initial recovery phase” – Pt 80* *“Consistency between physios” – Pt 4*
**Driving**	*n* = 3	*“More on driving - unsure if I can or not” – Pt 53*

### Readiness and recovery

Of the 35 pre-operative patients, 25 (71%) did not have any questions on the day of their procedure and 30 (86%) felt the information they had been given was tailored to them and their procedure. Forty-one (91%) of the 45 post-operative patients indicated they were confident they knew what was involved for them to recover and 44 (98%) felt ready for their procedure on the day.

### Other comments

The questionnaire ended with “Do you have any other comments about the information you were given that could be improved?” Seventeen of the 80 patients discussed the resources given to them here. Fifteen of these patients requested a single resource, either a webpage or a mobile application for their information needs. One suggested using E-learning modules for patients and another ended with the phrase “get into the digital age”, highlighting their frustration with paper. Six patients mentioned delays before being diagnosed and treated. A further patient admitted to not remembering much after the anaesthetic and was confused, pertaining to recovery and exercises, at home.

## Discussion

The primary aim of this project was to evaluate patients’ satisfaction with the information received during their operative experience and propose improvements. In general, patients were satisfied with the information and care they received. Some resources were found more useful than others and leaflets do not appear to meet the needs of the patient population anymore. Very few patients received or viewed webpages pertaining their procedure, but it was clear that many patients would value this. Exposing patients to other individuals who had undergone the same operation had a variable impact. Patients have reported that they want more specific and succinct information that applied to them and the procedure they are undertaking. Further information was requested on the topics of post-operative pain, recovery, the procedure and who would be in theatre with them.

### Resources

Many patients requested more internet resources, specifically asking for webpages about their procedures, online recovery timelines and a method of leaving questions for the surgeons to respond to in due course. A previous study has demonstrated that online resources can improve both the knowledge of patients and satisfaction with their surgery but should not be used in place of interactions with healthcare providers [23]. Another study suggested that all patients be directed to online resources, which would augment their knowledge and improve their informed status when consenting for procedures [24]. This suggests it may be worth investing in electronic resources to engage patients more. Such resources could take the form of expert patient blogs or vlogs, as many of the participants requested a balanced one to one meeting with a patient who had undergone the same procedure. With considerations around the logistics of organising meetings, including cost, infection risk and patient safety, expert patient blogs or vlogs would provide a regulated and cost-effective method of hearing about others’ experiences, proven to work in medical education, without the added cost and administration associated with in-person meetings or the bias observed on social media currently [25, 26].

Additionally, although patients undergoing replacement operations received the most literature and support from physiotherapists, they were less satisfied than the HTO patients, who currently do not receive any leaflets (one is in development) or group sessions at all. This could be due to bias from the surgeons, who are more enthusiastic about these procedures and may spend more time explaining the process and outcomes of the operation, subsequently improving the patient’s perception of their care and ensuring more accurate post-operative expectations [13, 14].

The variability of the results demonstrates that patients have differing values, highlighting holistic and patient centred care is vital in surgery and that the WHO’s integrated care model is important [21]. Resources must be of high quality, pitched at the right health literacy level and given to patients at the right time in their surgical experience to be of maximal benefit [27, 28]. The project also demonstrated that patients need different resources pre and post-operatively, reinforcing that tailored patient information, pain management and the use of other mediums such as social media and the internet may be useful in improving patients’ understanding of procedures and recovery [29, 30].

### Information topics

Common additional information requests were similar to other studies, and included post-operative activity levels, recovery, returning to work, how to cope at home and pain relief, including side effects [29, 31, 32]. A simple solution to this could be the development of a “Frequently Asked Questions” section on a website, whereby patients could seek out the answers to questions they have as they think of them.

Although the majority of current literature has not evaluated patient satisfaction of information, a previous study suggested that the NHS provide varied, conflicting and unstandardised literature to patients, concluding that patient involvement in the designing of resources is important [31]. Additionally, key pieces of information about a patient’s recovery are often given directly after they have been aroused from anaesthesia [31]. This poses problems as patients' memory function is impaired directly after surgery [33]. By delaying information provision until right before the patient is discharged, or at least 40 min after waking up from anaesthetic, retention could be drastically improved [33]. Better still, by giving out information packs in advance of procedures, surgeons could offer consolidated and pre-organised information for the patient to assimilate more easily and allow for a more informed decision making process.

### Patient involvement

Post-operative patients were statistically more likely to be dissatisfied with the information and care they had received, when compared to post-operative patients. Although there is no literature suggesting why this might be, it could be because patients are unsure what they want before their operations, or that we do not include what they would have found useful in the resources we give to them. Equally, most patients were questioned in the three to six-week window post-operatively, the period in which patients have the most pain without drastic functional benefit. A recurring theme throughout previous literature is the importance of engaging patients in the production of resources, but this is something rarely done in clinical practice [31]. A review around this topic suggested that patients’ coping strategies differ and employing needs based information provision, where patients are asked what information they might like to receive prior to a consultation, would enhance satisfaction and reduce pre-surgical anxiety [34]. An implication of this finding is that patients should actively be involved in designing materials for other patients, at least in the drafting phase. This should also be a continual evaluative process, whereby patients are asked to evaluate their functional outcomes as well information they received routinely. This could ultimately improve patient readiness, outcomes and satisfaction with the service provided.

### Strengths & weaknesses

Strengths of this project included the addition of free-text answers in the questionnaire so patients could reflect on what they felt was important for us to know. The project also evaluated a range of surgeons, evaluating most of the service. Limitations include the sample population size being relatively small and not ensuring that all patients answered the same questions. Additionally, there was an unequal distribution of procedure types and ages, meaning some groups may be under- or over-represented, which limited the depth of analysis we could perform within subgroups. Post-operative patients were interviewed up to a year after their operation, meaning that some had forgotten about the specifics of their procedure, potentially enabling recall bias. Finally, the same patients were not interviewed before and after their procedures. This means that comparison between post and pre-operative patients should be interpreted with caution, although having a variety of patients allows more voices to be heard throughout the process. Moreover, using a thematic analysis is relatively subjective and although all effort was made to reduce bias, this is difficult to eradicate from a qualitative methods analysis.

## Conclusion

In this exploratory study, patients were generally satisfied with the information and care they received but not all elements of the information were found useful. This could be improved by the production of webpages that include educational videos, patient experiences and an interactive recovery timeline or by developing expert patient blogs to aid clarity of post-operative expectations. Other proposed electronic resources include a mobile information platform that is tailored to each procedure, that can ask healthcare professionals questions or give access to FAQs that patients can refer to when the time is right. Patient involvement in the development and delivery of resources would be worthwhile and would enhance the resources developed. These suggestions may enable NHS Trusts to “get into the digital age”, however, more research on patient satisfaction around information provision and the impact it has on recovery as well as decision making is needed.

## Supplementary Information

**Additional file 1.**

## Data Availability

The datasets used and/or analysed during the current study are available from the corresponding author on reasonable request.

## Abbreviations

ACLR: Anterior cruciate ligament repair
FAQ: Frequently asked questions
HTO: High tibial osteotomy
NHS: National Health Service
PRO: Patient reported outcomes
THR: Total hip replacement
TKR: Total knee replacement
WHO: World Health Organisation
