# Supplementary material for: A patient satisfaction survey investigating pre- and post-operative information provision in lower limb surgery
Source: BMC Musculoskelet Disord. 2020 Nov 16;21:754. doi: 10.1186/s12891-020-03761-w (PMC7667776; doi:10.1186/s12891-020-03761-w)
Supplement: Supplementary file 1 — Additional file 1. [file 12891_2020_3761_MOESM1_ESM.pdf]

How satisfied were you with the information you got before and after your surgery?

Some patients have noticed that they are not entirely satisfied with the information they are given before and after their operations and may have had unanswered questions before their procedures.

We invite you to complete a short survey to ask you about information given to you before and after your procedure to identify any potential improvements we could make to your experience. It should take no longer than 15 minutes to complete. It can be done on a mobile device, but may be easier to complete on a computer. Printed versions are also available.

This project aims to evaluate information provision to a population of patients undergoing lower limb surgery to identify if and how the type, method and matter of information can be changed to improve satisfaction.

Please fill out *either* the Pre-operative information section if have not had your operation yet, *or* the Post-operative section if you have already had your operation.

Privacy Notice

Any information collected will not be able to identify you and will not be combined with other information in a way that could identify you. The information will only be used for the purpose of this service evaluation, and cannot be used to contact you, affect your care, or future experiences. It will not be used to make decisions about future services available to you.

If you have any concerns or queries, please contact a member of the research team via the email address below:

Max Renna - email: m.renna@warwick.ac.uk

Should you wish to withdraw your information from this study, please do not submit the questionnaire as data cannot be identified once you have submitted.

Before we start, we need to ask some questions about you.

What is your Gender?

- ○ Male
- ○ Female
- ○ Prefer not to say
- ○ Other

What is your age?

- ○ 20 or younger
- ○ 21-30
- ○ 31-40
- ○ 41-50
- ○ 51-60
- ○ 61-70
- ○ 70+

What procedure are you having/did you have?

- ○ Knee replacement
- ○ Hip replacement
- ○ Cruciate ligament repair
- ○ Achilles tendon repair
- ○ Meniscus repair
- ○ Neck of femur repair
- ○ Other (Please specify) ______________________________

Pre-operative information

Here, we would like to ask you about the information you received prior to your operation and would like to hear about ways in which we could improve the experience for other patients, like yourself, who need the procedure.

How satisfied were you with the information you received before your procedure? (Please Circle)

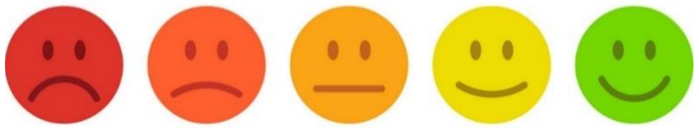

Extremely dissatisfied — Extremely Satisfied

Please tell us how useful these pre-operative sources of information were:

	Extremely useful	Useful	Not useful	Very unhelpful	Did not receive/use
Leaflets from hospital	○	○	○	○	○
Internet webpages	○	○	○	○	○
Discussions with your doctor	○	○	○	○	○
Apps on your phone	○	○	○	○	○
Friends and Family	○	○	○	○	○
Other	○	○	○	○	○

Please tell us more about why you choose the responses you gave

____

____

____

If you used internet resources, which ones did you use?
(Select all that apply)

- [ ] NHS website
- [ ] Hospital website
- [ ] Patient.info
- [ ] Nuffield health
- [ ] BUPA health
- [ ] WebMD
- [ ] Other American website (please specify) ____
- [ ] Other UK website (please specify) ____

Which resource did you find most useful? and why?

____

____

Did you have any unanswered questions before your operation?

◯ Yes (1)

◯ No (2)

Please list the questions you had (if any) below:

____

____

____

____

Did you feel that the information given to you was tailored to your procedure?
Tailoring includes things such as having leaflets that were for appropriate for your age group, video guides that mentioned goals related to you or any other way of making the resources feel personal to you.

- ○ Yes
- ○ No

What topics would you have liked to have received more information on, before your procedure? (Select all that apply)
Here, we would like to identify any areas that weren't addressed in enough detail during the pre-operative phase of your procedure

- ☐ Post-operative pain
- ☐ The procedure
- ☐ Your condition
- ☐ Who will be in the operating room with you (staff, students and company reps)
- ☐ How long it will take to recover
- ☐ How long before you can go back to work
- ☐ Where you needed to be and when
- ☐ Other ______________________________

Please tell us any other information you would have found useful to know before your operation:

____

____

____

We have thought of a few ways to improve the service we provide, please tick those that would help you, if you had the procedure again:

- ☐ Leaflets
- ☐ 1 to 1 with a healthcare professional
- ☐ Group session with other patients
- ☐ 1 to 1 with another patient who has had the procedure
- ☐ App on phone
- ☐ Internet page
- ☐ Other (please specify) ______________________________

Please tell us more about your choice

____

How satisfied have you been so far with the care you have received so far? (Please circle)

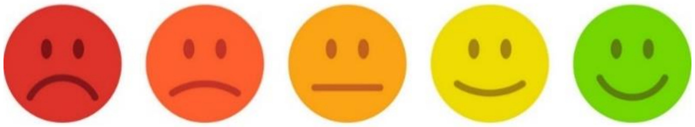

Extremely dissatisfied — Extremely Satisfied

Do you have any other comments about the information you were given before your operation that could be improved?

____

____

____

____

____

Post-Operative information

Here, we would like to know more about what information you received after your operation and identify if it met your expectations, exceeded them or did not meet them at all. The questions relate to aspects of your recovery once you had your procedure performed.

How satisfied were you with the information you got after your operation? (Please Circle)

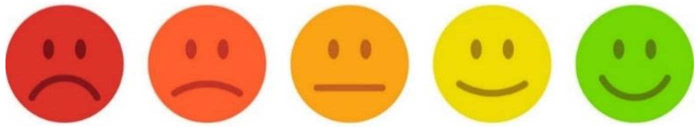

Extremely dissatisfied — Extremely Satisfied

Did you feel ready for your procedure before you had it?

- ○ Yes ____
- ○ No ____

How did you receive information after your operation?
Here, we would like to know if you received any information after your operation.

- ☐ Leaflet
- ☐ Discharge letter
- ☐ Discussion with surgeon
- ☐ Discussion with nurse
- ☐ Letter
- ☐ Webpage you could visit
- ☐ Phone number in case you had any problems
- ☐ Other (please specify) ____

Which options above would you have liked to have access to after your operation?

- [ ] Leaflet
- [ ] Discharge letter
- [ ] Discussion with surgeon
- [ ] Discussion with nurse
- [ ] Letter
- [ ] Webpage you could visit
- [ ] Phone number in case you had a problem
- [ ] Other (please specify) ______________________________

Please tell us how useful the post-operative sources of information, that you used, were:

	Extremely useful	Useful	Not useful	Very unhelpful	Did not receive/use
Leaflets from hospital	○	○	○	○	○
Internet webpages	○	○	○	○	○
Discussions with your doctor	○	○	○	○	○
Apps on your phone	○	○	○	○	○
Friends and Family	○	○	○	○	○
Other (please specify)	○	○	○	○	○

Did you feel ready to go home when you were discharged?
Some patients who come in for the day may not feel ready to go home at the end of the day. Even if you spent a longer or shorter period of time in hospital, please tell us if you felt ready to go home or not. This could include unanswered questions or not having enough support at home.

____

____

____

____

____

Did you feel confident you knew what was involved in order for you to recover from your procedure?
It can be difficult to remember everything you get told after an operation, if you did or didn't feel confident on what you able to do and how long after you operation you could start moving around, please tell us.

- ◯ Yes
- ◯ No

How satisfied were you with the information given to you about the topics listed below?

	Extremely satisfied	Slightly satisfied	Neither satisfied nor dissatisfied	Slightly dissatisfied	Extremely dissatisfied	Did not receive
Returning to your normal activities	○	○	○	○	○	○
Pain medication	○	○	○	○	○	○
How much pain you would be in	○	○	○	○	○	○
How long to recover	○	○	○	○	○	○
How to care for your wounds	○	○	○	○	○	○
Support available	○	○	○	○	○	○
Other (Please specify)	○	○	○	○	○	○

Was there a specific area of your recovery you would have liked more information on?
Sometimes doctors can forget to tell you things or you don't have a question until it's too late and you are struggling to do something. Tell us if there was an area you wanted more information on.

____

____

____

____

____

If you were to have this procedure again, is there anything about the information you were given that you would like to change?

How satisfied are you with the level of care you received? (Please Circle)

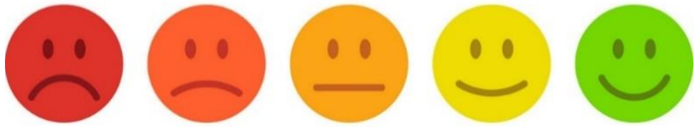

Extremely dissatisfied

Extremely Satisfied

Is there anything else you think might be useful for us to know to improve our service in the future?
